# Dual-Agent Photodynamic Therapy with Optical Clearing Eradicates Pigmented Melanoma in Preclinical Tumor Models

**DOI:** 10.3390/cancers12071956

**Published:** 2020-07-18

**Authors:** Layla Pires, Valentin Demidov, Brian C. Wilson, Ana Gabriela Salvio, Lilian Moriyama, Vanderlei S. Bagnato, I. Alex Vitkin, Cristina Kurachi

**Affiliations:** 1 São Carlos Institute of Physics, University of São Paulo, Sao Carlos-SP 13566-590, Brazil; layla.pires@uhnresearch.ca (L.P.); lili@ifsc.usp.br (L.M.); vander@ifsc.usp.br (V.S.B.); cristina@ifsc.usp.br (C.K.); 2 Department of Medical Biophysics, University of Toronto, Toronto, ON M5G 1L7, Canada; val.demidov@mail.utoronto.ca (V.D.); alex.vitkin@rmp.uhn.ca (I.A.V.); 3 Princess Margaret Cancer Centre, University Health Network, Toronto, ON M5G 2C1, Canada; 4 Amaral Carvalho Cancer Hospital, Jau-SP 17210-070, Brazil; gasalvio@hotmail.com

**Keywords:** melanoma, photodynamic therapy, optical clearing agent

## Abstract

Treatment using light-activated photosensitizers (photodynamic therapy, PDT) has shown limited efficacy in pigmented melanoma, mainly due to the poor penetration of light in this tissue. Here, an optical clearing agent (OCA) was applied topically to a cutaneous melanoma model in mice shortly before PDT to increase the effective treatment depth by reducing the light scattering. This was used together with cellular and vascular-PDT, or a combination of both. The effect on tumor growth was measured by longitudinal ultrasound/photoacoustic imaging in vivo and by immunohistology after sacrifice. In a separate dorsal window chamber tumor model, angiographic optical coherence tomography (OCT) generated 3D tissue microvascular images, enabling direct in vivo assessment of treatment response. The optical clearing had minimal therapeutic effect on the in control, non-pigmented cutaneous melanomas but a statistically significant effect (*p* < 0.05) in pigmented lesions for both single- and dual-photosensitizer treatment regimes. The latter enabled full-depth eradication of tumor tissue, demonstrated by the absence of S100 and Ki67 immunostaining. These studies are the first to demonstrate complete melanoma response to PDT in an immunocompromised model in vivo, with quantitative assessment of tumor volume and thickness, confirmed by (immuno) histological analyses, and with non-pigmented melanomas used as controls to clarify the critical role of melanin in the PDT response. The results indicate the potential of OCA-enhanced PDT for the treatment of pigmented lesions, including melanoma.

## 1. Introduction

Melanoma arises from the melanocytes and is highly invasive and metastatic. According to the American Cancer Society, melanoma rates have been rising for the last 30 years and are expected to increase by 2% in 2020 [1], with about 100,350 new melanoma cases and 6850 deaths in the USA alone. Surgical resection is the standard first-line treatment for primary melanoma, often combined with chemotherapy, radiation therapy and, more recently, immunotherapy such as with the use of anti-PD1 monoclonal antibodies [2,3]. However, local recurrence rates remain high (≈40%) [4] and mortality rates have not improved significantly in the past 20 years.

Most melanomas (≈96%) are highly pigmented [5], which negatively impacts light-based treatments. Photodynamic therapy (PDT), using light-activated compounds (photosensitizers) to produce reactive cytotoxic species such as singlet oxygen, is already established for non-melanoma skin cancer, particularly basal cell carcinoma in which it has >90% cure rate in non-nodular lesions [6]. However, reported PDT responses in melanoma have been poor to date [7]. Several strategies have been investigated in preclinical cell and animal models to improve the efficacy, including combining PDT with tumor suppressors [8] or anti-angiogenic drugs [9], the use of tumor-targeted photosensitizers [10] or nanoparticles [11], photosensitizer cocktails [12] and, especially, the use of new photosensitizers activated at longer near-infrared wavelengths where the melanin absorption is lower [13]. For example, Methylene blue-PDT showed enhanced tumor cell apoptosis and animal survival in an in vivo murine model compared to untreated controls, but full-thickness tumor destruction was not achieved [14]. The use of a vascular-targeted photosensitizer, WST11, activated at long-wavelength (≈775 nm) in a mouse pigmented-melanoma xenograft model using transcutaneous illumination immediately after photosensitizer injection showed tumor necrosis, followed by tumor flattening in the first 14 days and an encouraging 70% cure rate at 90 days using optimal photosensitizer and light doses [15]. Nanoparticle formulations have shown promising PDT outcomes in immunocompetent mice, with an increase in tumor specificity and ROS generation: after near-infrared light activation, tumor control and survival up to 1year post treatment have been reported [16]. 

Baldea et al. [7] and Huang et al. [17] reviewed PDT studies in melanoma and identified four potential contributors to the poor responses: (1) the high absorption of melanin that attenuates the treatment light and confines tumor kill to only superficial depths; (2) the anti-oxidant properties of melanin that block the cytotoxic reactive-oxygen photoproducts; (3) sequestration of photosensitizer within the melanosomes that reduces free-radical generation within the tumor cells, and (4) efflux of photosensitizer by active transporters. The first three factors are unique to melanoma. They proposed, therefore, that an optimal PDT treatment should involve the direct tumor-cell photo damage, destruction of the tumor vasculature, and activation of immunological responses. Here we address the light penetration barrier through optical clearing of the tissue. Additionally, the second and the third factors are circumvented by using a dual-photosensitizer strategy, wherein one photosensitizer targets primarily the cellular compartment of the tumor, and the second acts primarily through destruction of its microvasculature. 

In the optical diffusion regime, the effective penetration depth, i.e., the depth, δ, at which the light fluence drops to 1/e of its initial value, is given approximately by $\delta =1/\sqrt{3\mu_{a}\mu {’}_{s}}$, where $\mu_{a}$ and $\mu {’}_{s}$ are the absorption and reduced scattering coefficients of the tissue. That is, the absorption and scattering both contribute to the light attenuation. Garcia-Uribe and collaborators [18] measured the optical absorption and reduced scattering coefficients of melanomas as, respectively, ≈0.3 and ≈18 cm^−1^ in the 670–690 nm wavelength range. The latter was significantly higher than in any other lesions, ascribed as being due to the atypically large nuclei [18]. Thus, optical attenuation in pigmented melanomas is due not only to its high pigmentation (absorption) but also to its high scattering properties. Hence, one method to improve light penetration is to use optical clearing agents, which are hyperosmotic compounds that displace tissue water to reduce the optical scattering by reducing refractive-index mismatch at cell interfaces. Its effect has been demonstrated by confocal microscopy in ex vivo skin samples [19] and by improved spatial resolution at depth of optical coherence tomography (OCT) in normal skin [20]. We have also recently demonstrated optical clearing agent (OCA)-induced doubling of the effective depth of microvascular OCT imaging in a pigmented cutaneous melanoma murine model, which was used also in the present study [21]. The clearing process is attractive clinically, as the effect is reversible once the agent leaves the tissue and no significant toxicity from topical application has been reported. Depending on the OCA, optimal clearing occurs after about 15 min [21]. Hydrophilic polyethylene glycol (PEG)-based polymers and lipophilic polypropylene glycol (PPG)-based polymers are approved by the FDA for medical applications [22]. These compounds both have a refractive index ≈1.47, close to that of dermal collagen [23]. 

Two hypotheses were tested here: (1) that using a combination of cellular-PDT with Photodithazine (PDZ) and vascular-PDT with Visudyne (VIS) improves the treatment response in melanoma compared to single-agent PDT, and (2) that topical application of OCA immediately before treatment improves the PDT efficacy sufficiently to enable complete tumor destruction up to about 1 mm thickness, which corresponds to Clark level II melanoma [24]. Treatments were carried out in vivo using pigmented cutaneous murine-derived melanoma grown intra-dermally in nude mice. Non-pigmented melanoma served as PDT response-positive controls. Intravital ultrasonography and photoacoustic imaging of tumor volume and post-mortem (immuno) histology were used to assess the PDT treatment responses. In addition, speckle-variance optical coherence tomography, svOCT, was performed in vivo in a separate dorsal-window-chamber model of melanoma to directly visualize the microvascular treatment response. To our knowledge, this is the first report of the combination of optical clearing and photodynamic therapy for any tumor, and the first report of full-thickness tumor eradication of pigmented melanoma in an in vivo immunocompromised animal model using commercially available photosensitizers. Moreover, this is the first study to investigate the same PDT protocol in both pigmented and non-pigmented melanoma models, induced with melanotic and amelanotic cloned cells, to demonstrate clearly the effect of melanin in the PDT outcome.

## 2. Materials and Methods

All animal procedures were approved by institutional Animal Care Committees (University Health Network, Toronto, ON, Canada, protocols 4401.1 and 3256.7, and São Carlos Institute of Physics, University of São Paulo, Brazil, protocol 03/2014).

### 2.1. Optical Clearing Agent

PEG-400 and 1,2 propanediol were purchased from Sigma-Aldrich (USA). A 19:1 mix was prepared using PEG-400 and the transdermal permeation enhancer 1,2-propanediol, as described by Wen et al. (2012) [25].

### 2.2. Cutaneous Melanoma Model 

B16F10 pigmented murine melanoma cells were purchased from American Type Culture Collection (ATCC, Manassas, VA, USA). B78H14 non-pigmented murine melanoma cells were kindly donated by Dr. Pier-Luigi Lollini, University of Bologna, Italy. Both cell lines were cultured in Dulbecco Modified Eagle’s Medium supplemented with 10% fetal bovine serum and antibiotics at 37 °C and 5% CO_2_. Cutaneous melanoma was induced by intradermal injection of 10^5^ cells in one flank of adult female nude mice (J: NU homozygous, Jackson Labs) using a 30ga needle under inhaled anesthesia (isoflurane 5% for induction and 2% for maintenance). Animals were kept in micro-isolator cages with access to food and water ad libitum. 

### 2.3. PDT Treatment 

Two different photosensitizers were used, either singly or in combination: Photodithazine (VETA GRAND, Russia) to target primarily the tumor cells and Visudyne (Novartis, Toronto, ON, Canada) for vascular-mediated treatment. The treatment groups are summarized in Table 1. Treatments were initiated when the Breslow depth, i.e., the distance from the skin surface to the deepest layer of the tumor, reached 0.9–1.1 mm (tumor thickness 0.8–1.0 mm), determined as described below. The tumors were 3–5 mm in surface diameter. Under general anesthesia (5% isoflurane for induction, 2% for maintenance) the photosensitizer was administered into the tail vein at a concentration of 1.0 mg/kg body weight for Photodithazine and 0.8 mg/kg for Visudyne (based on pilot studies). These concentrations were halved for dual-agent treatment. The photosensitizer-light time intervals were 15 min for Visudyne (based on vascular endothelial cell uptake) and 60 min for Photodithazine (biased towards tumor cell uptake). The mice were kept under low ambient light after photosensitizer administration. For optical clearing, following earlier studies [21], OCA (≈300 μL) was applied topically 15 min before light irradiation and re-applied if drying of the tissue surface was evident during treatment. Prior tape stripping was used to remove the stratum corneum [26] to improve the OCA penetration into tumors. The light parameters were set to prevent any possible thermal effect [27] and were also based on previous clinical trials [28] and preclinical models [29]. With Photodithazine, light from a 670 nm diode laser was delivered in a 1 cm diameter spot encompassing the tumor at an incident irradiance of 100 mWcm^−2^ for 1000 s, giving an energy fluence of 100 Jcm^−2^. A 690 nm diode laser was similarly used to activate Visudyne at 80 mWcm^−2^ and 80 Jcm^−2^. With the combined photosensitizers, the energy fluences were reduced to 60 and 40 Jcm^−2^, respectively, and Photodithazine-PDT was given first, followed immediately by Visudyne-PDT. The fluences were reduced compared to the single treatment to minimize the off-target effects, such as inflammation, erythema and pain. This sequence was chosen to avoid reducing the tumor oxygenation due to the vascular shutdown following VIS-PDT that could compromise the direct tumor-cell cytotoxicity of PDZ-PDT. Buprenorphine (0.1 mg/kg) was administered for analgesia before and then every 8 h for 72 h after treatment. 

### 2.4. Treatment Response Evaluation 

The tumor volume was measured using 3D ultrasound scanning on a Vevo 2100 system (FUJIFILM VisualSonics Inc. Canada) operating at 32–56 MHz, with the images analyzed using ImageJ software (NIH, Bethesda, MD, USA). This instrument also incorporated a tunable pulsed laser (680–970 nm, 20 MHz pulse repetition rate) for photoacoustic imaging at 700 nm that served primarily to confirm the presence and absence of melanin in the pigmented and non-pigmented tumors, respectively, and to complement the ultrasound measurements. The tumor volume was measured immediately before treatment and then every 48 h until the sacrifice by cervical dislocation under general inhaled anesthesia, either at 10 d after treatment or earlier if humane endpoints (excessive lethargy, hunched or abnormal posture, tumor ulceration) occurred or if the tumors grew to >10 mm diameter. Sacrifice at 10 d post-treatment was chosen to allow histological analyses and quantification of S100 and Ki67 biomarkers. Moreover, we used immunocompromised animals specifically to avoid any immune-regulation component of the PDT response and, since the tumors were imaged every 48 h to track the change in volume accurately and longitudinally over time, this required procedures outside the biosafety environment, with the corresponding risk of infection for these immunocompromised animals. Studies with longer follow up in immunocompetent mice are in progress. 

The specific growth rate (SGR) and doubling time (DT) were calculated as in Mehrara et al. [30], using the volumes (V) at each time point (t) and fitting the growth curves with single exponentials, as in Equations (1) and (2). The calculations were performed only in those experimental groups having detectable tumor remaining at 10 d post-treatment.

(1) $$SGR=\frac{ln\left(\frac{V_{2}}{V_{1}}\right)}{t_{2}-t_{1}}$$

(2) $$DT=\frac{ln2}{SGR}$$

### 2.5. Histology Analysis

Upon sacrifice, the tumors were excised and processed for (immuno) histology. Tissue sections (5 μm thick) cut perpendicular to the surface and showing all layers of skin and tumor (top to bottom) were stained with H&E and immunostained for S100 (1:500 Z-0311; Dako–Agilent Technologies, Santa Clara, CA, USA) and Ki67 (1:500 GA626; Dako–Agilent) expression as markers of the presence of melanoma cells [31], and cell viability and proliferation [32], respectively. WARP-Red chromogen, an alkaline phosphatase system, was used to distinguish between the positive-stained cells and melanin pigmentation. Two sections from each tumor were stained and digitized (Aperio ImageScope^®^; Leica, Canada) and the images were processed using commercial software (HALO^®^, Indica Labs) to measure the tumor area and total tumor cell count and to quantify the specific protein-expression levels. Immunostaining was considered negative when <10% of tumor cells showed positivity. The S100-stained sections were used to measure the largest tumor thickness, with three measurements being performed in each section. 

### 2.6. Statistical Analysis

The immunohistological and tumor-depth results are presented as mean values ± standard deviation, and statistical comparisons were performed to test differences between the treatment groups and untreated controls, as well as between treatment groups using one-way analysis-of-variance (ANOVA) with post-hoc comparisons with the Tukey test (GraphPad Prism 7 software; GraphPad Software, Inc., San Diego, CA, USA): *p* < 0.05 was considered statistically significant.

### 2.7. PDT Vascular Effects 

Additional experiments were performed to image directly the effect of PDT on the tumor vasculature using a dorsal-window-chamber model, as described previously [33]. Briefly, 10^6^ cells were injected intradermally into the dorsal skin of nine mice (*n* = 3 per group) and a window chamber was installed 3 days later to allow direct visualization of the tumor. The tumor vasculature was assessed 2 days later by speckle-variance optical coherence tomography (svOCT) to visualize subsurface tissues with sensitivity to capillary-level microvasculature and micron-scale resolution. PDT was performed through the chamber window using the same photosensitizer and light doses as in the intradermal tumor model. The microvascular response was assessed at 2, 24, and 48 h and 7 d post-treatment. In this model, only the vascular response to PDT without optical clearing was investigated, since adding OCA risked compromising the window preparation. Moreover, the effects on melanoma of OCA alone has been previously reported by us [21]. 

The OCT system was based on a quadrature Mach-Zehnder interferometer with optical amplification [34], incorporating a 1320 nm light source (HS2000-HL, Santec, Komaki, Japan) and 20 kHz polygon-based tunable filter, with a sweeping range of 110 nm and average output power of 10 mW. The axial and lateral resolutions in air were 8 and 15 μm, respectively. Volumetric microangiography (field of view 6 × 6 × 1 mm) was performed in speckle-variance mode [35], capturing eight B-model OCT images from the same location with a 25 ms time inter-frame delay while scanning laterally. This technique yields images of the vasculature, based on the detection of red blood cell motion, and SV contrast of 10% above the noise floor was defined as microvascular blood [35]. For visual representation, the images were depth-encoded in RGB color space [36] using Matlab software (Mathworks, Natick, MA, USA) with 256 levels. 

## 3. Results

### 3.1. Window Chamber Model Responses 

**Figure 1:**
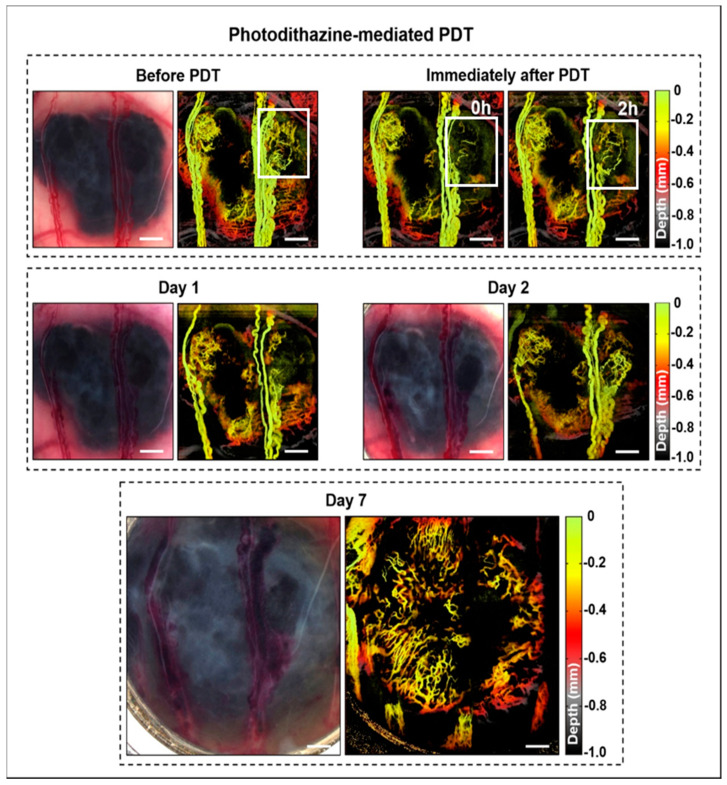
Example of microvascular images immediately before and at different times after Photodithazine (PDZ)-PDT. These are en-face images, color-coded for depths in the tumor. Changes in the tumoral micro vessels immediately after PDT are highlighted by the white square. At 2 h post-PDT the vascular network was re-established, and tumor growth continued until day 7.

The svOCT images demonstrated the rich vascular network around and within the tumors before treatment. As seen in Figure 1, PDZ-mediated PDT affected only the smaller vessels, which was observable immediately after treatment, with tumor regrowth and angiogenesis seen at 24 h later. 

**Figure 2:**
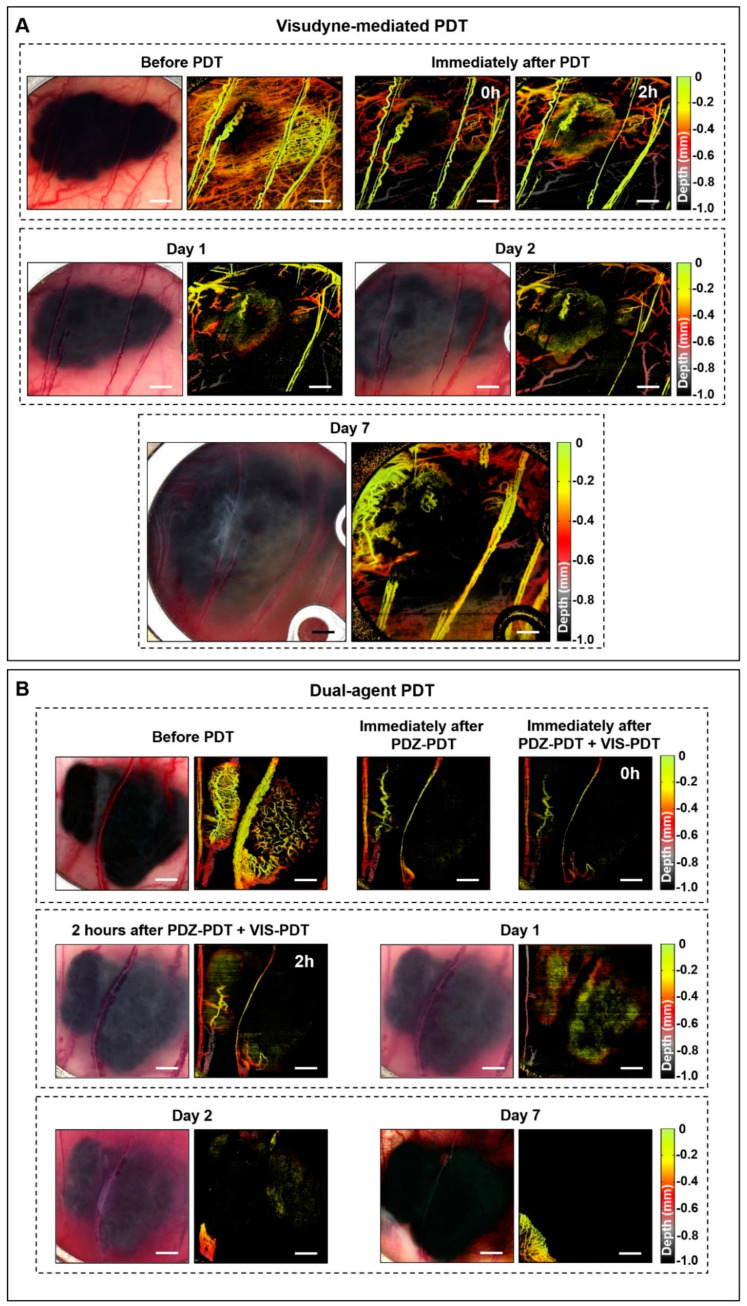
Optical coherence tomography (svOCT) angiographs of pigmented melanoma responses to (**A**) vascular-PDT with Visudyne (VIS) and (**B**) dual-agent PDT. In (**A**) marked vascular damage is observed immediately after PDT. Larger feeder vessels were partially damaged but were re-established by day 7. In (**B**) the vessels were largely destroyed after PDT and blood leakage was observed throughout the tumor on day 1. No vascular regrowth was observed on day 7.

PDT with Visudyne at a short drug-light interval of 15 min (as used clinically for age-related macular degeneration to destroy abnormal choroidal neovasculature [37]) was more effective in shutting down the vasculature than PDZ-PDT, leading to marked tumor growth delay: Figure 2A. However, regrowth was seen within 7 d post-treatment. The combined treatment with PDZ-PDT plus VIS-PDT resulted in immediate small-vessel closure from the first photosensitizer, with large-vessel responses with the subsequent VIS-PDT, so that there was complete vascular shutdown at 2 h post-treatment: Figure 2B. Unlike the single-agent PDZ-PDT treatment, there was no recovery of the vasculature within the 7 days observation period. Thus, the dual-photosensitizer strategy appears effective in this pigmented melanoma model. Due to the near-2D tumor morphology in the window chamber, the effect of light attenuation is less than in the solid 3D intradermal tumors.

### 3.2. Intradermal Melanoma Responses

**Figure 3:**
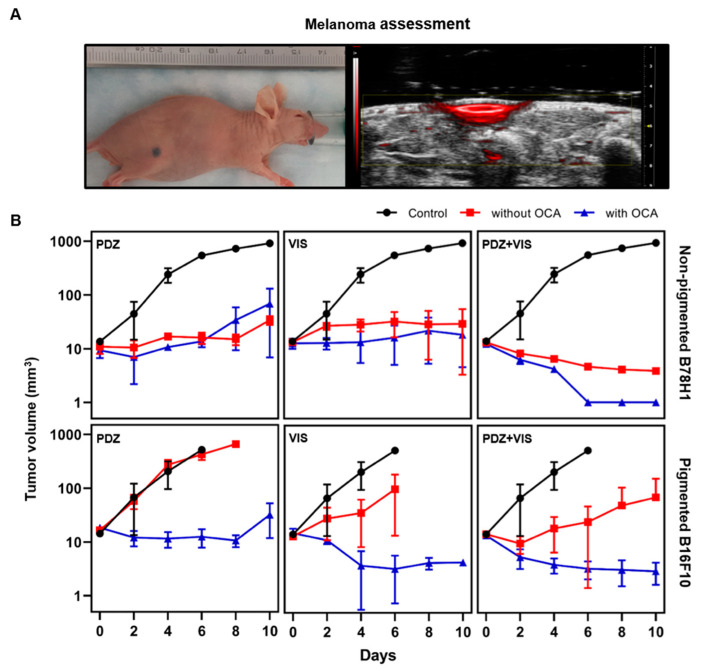
Cutaneous melanoma model and tumor volume assessment. (**A**) Example of a pigmented melanoma and corresponding hybrid ultrasound (greyscale) and photoacoustic (red) image. (**B**) Tumor volume (mean ± 1 standard deviation) with and without optical clearing in pigmented and non-pigmented tumors following PDT on day 0. *n* = 3 for both single-agent PDT cohorts and *n* = 5 for dual-agent PDT.

The use of both pigmented tumors and non-pigmented melanoma controls allowed factors such as photosensitizer uptake and intrinsic photodynamic effect, both of which are clinically important parameters that are independent of the presence of melanin, to be distinguished from melanin-dependent antioxidant and light absorption/scattering factors. An example of a pigmented tumor and corresponding ultrasound/photoacoustic images is shown in Figure 3A. There was no measurable difference in the pretreatment tumor growth rate between the pigmented and non-pigmented tumors.

The tumor volume assessment in the non-pigmented tumors showed that PDZ-PDT halted tumor growth for the first 6 days but regrowth was observed subsequently. VIS-PDT showed similar growth arrest, but this was maintained through day 10: Figure 3B. Not surprisingly in the absence of tumor pigmentation, optical clearing had no measurable impact on either photosensitizer. Continuous reduction of the tumor volume was seen after dual-photosensitizer PDT and, with the addition of optical clearing, no tumor was detectable at or beyond day 6. Histological assessment (see below) indicated that the residual volume detected in the group without optical clearing was completely necrotic.

By contrast, in the pigmented tumors the single-agent treatments produced only minimal to mild responses, with Visudyne being slightly more effective than Photodithazine: Figure 3B. Dual-photosensitizer PDT showed somewhat greater efficacy in terms of reduced tumor growth, but this improvement was much less than in the non-pigmented tumors. The optical clearing had a marked effect in both single- and dual-photosensitizer treatments, yielding tumor growth responses comparable to those in the corresponding non-pigmented groups. 

The volumetric specific growth rate (SGR) and tumor doubling time (DT) profiles are shown in Table 2. In non-pigmented tumors, single-photosensitizer PDT reduced the SGR by around 80%, with or without optical clearing. Dual-agent PDT yielded negative growth rates that were related to tumor remission, as confirmed by histology. In the pigmented tumors, no difference was observed between the untreated control and PDZ-PDT groups, indicating that cellular-PDT is largely ineffective, while growth-rate reductions of ≈50% and ≈90% were achieved in the VIS-PDT and dual-photosensitizer groups, respectively. The additional optical clearing resulted in negative growth rates for all treatment groups. 

The DT values showed the same general behavior. For the non-pigmented tumors, single-agent treatments increased the DT values by 2- to 8-fold over the untreated controls, corresponding to altered tumor growth and progression within the 10 d evaluation period. The doubling times for the dual-agent photosensitizer, with and without optical clearing, were negative, indicating tumor remission. In the pigmented tumors, no difference in doubling time was observed between single-agent PDT and untreated controls, but dual-agent PDT increased the DT value by ≈10-fold. With the addition of optical clearing, all treatment groups showed negative values of the doubling time.

**Figure 4:**
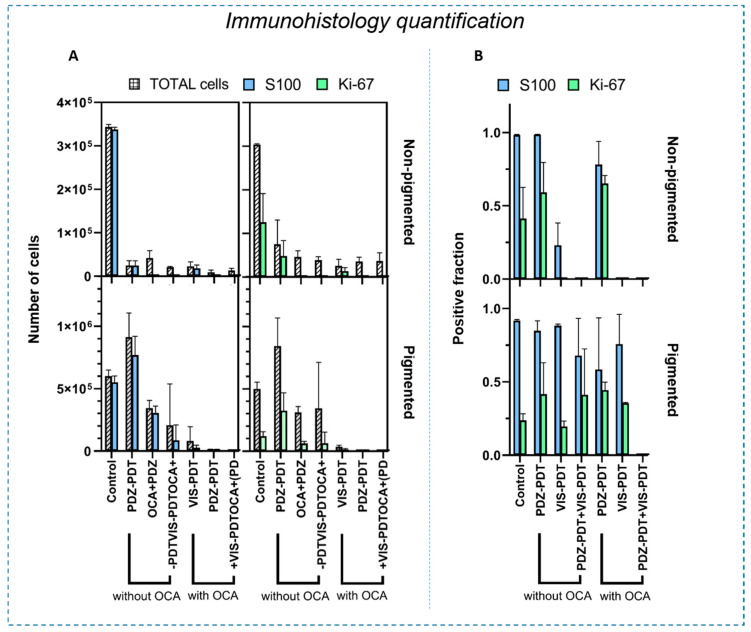
Quantification of S100 and Ki-67 expression at 10 d after PDT, comparing pigmented and non-pigmented tumors. (**A**) Each panel shows the total number of cells and the number of positively stained cells for each of the treatment protocols. (**B**) Corresponding positive fractions, i.e., the ratio of positively-stained cells to total cells. Error bars indicate ± standard deviation.

While the in vivo imaging was able to monitor the tumor volume (ultrasound/photoacoustic) and microvasculature (svOCT), it does not give information on the cellular response. Hence, this was assessed by quantitative histology in H&E, S100, and Ki67 staining of tissue sections. (Details of the histological analyses are presented in Figures S1–S4). The S100 and Ki67 protein expression levels are summarized in Figure 4. 

In the non-pigmented tumors, the treatment decreased the overall number of tumor cells in all groups. However, the PDZ-PDT group still showed 53% and 64% of the cells expressing Ki-67 with and without optical clearing, respectively. (The fact that these levels were lower in the non-treatment control group is an artifact due to the negatively-staining necrotic core that develops as the tumors grow). By contrast, none of the groups treated with VIS-PDT had detectable S100 or Ki-67 staining in the non-pigmented tumors (*p* < 0.0001 compared to untreated controls), indicating that targeting the vasculature was more effective than targeting the tumor cells directly; this is consistent with the fact that melanomas have a rich vascular network [38]. No difference was observed between VIS-PDT and dual-agent PDT in the non-pigmented tumors, independent of the use of OCA, confirming that light penetration was not a significant limiting factor. 

In pigmented tumors, VIS-PDT and dual-agent PDT showed a marked decrease in the number of cells compared to untreated controls, with and without clearing. However, considering the ratio of positively-stained cells to the total number of tumor cells, this decrease was likely related to the overall tumor size reduction and not to reduced tumor cell activity. In some groups, these data differed from the tumor volume measurements, which do not distinguish between viable and necrotic tumor. These results agree with other melanoma studies in which PDT could control the disease but not eradicate it. The addition of OCA increased the PDT efficacy in all treatment groups, as seen by the reduction in the total number of tumor cells. However, only the combination of optical clearing and dual-photosensitizer treatment resulted in the complete absence of S100 and Ki-67 staining, implying that no viable melanoma cells remained. 

**Figure 5:**
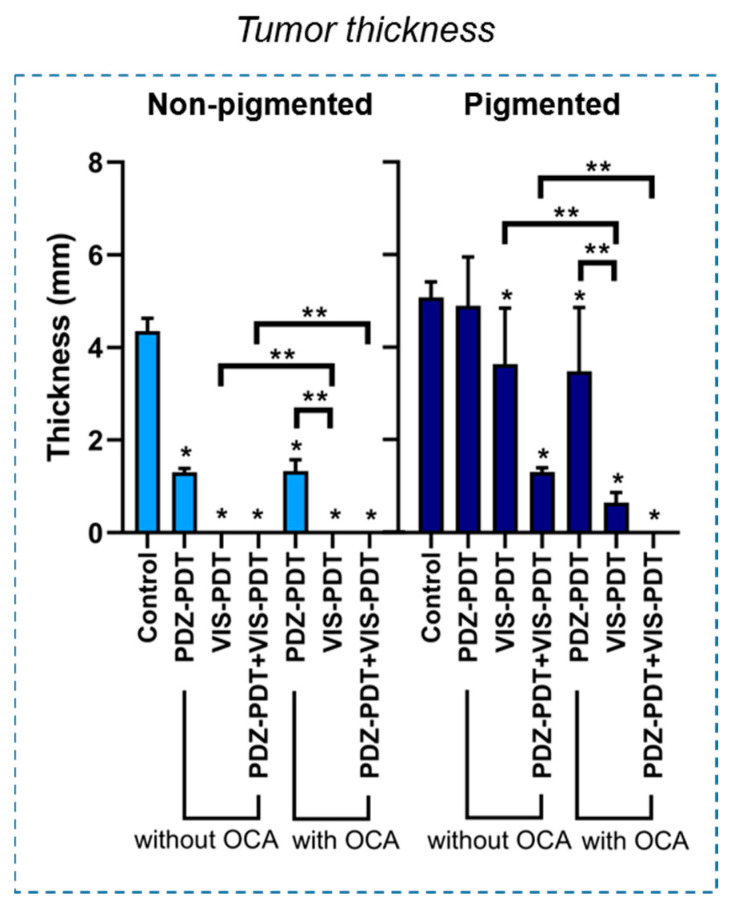
Thickness of the remaining tumor at the end of the evaluation period. * Statistical significance (*p* < 0.01) compared to the control group, ** statistical significance (*p* < 0.01) between treatment groups.

Figure 5 shows the thickness of the remaining viable tumor as measured on the S100-stained slides. In non-pigmented melanoma, all treatment groups were significantly different from the untreated controls (*p* < 0.0001). In these lesions, a reduction in tumor thickness of ≈65% was observed for PDZ-PDT, with no significant change by the addition of OCA. Correspondingly, no residual tumor was seen in either the VIS-PDT or dual-agent PDT groups, with or without optical clearing. By contrast, for pigmented melanoma VIS-PDT reduced the tumor thickness by ≈30% and ≈20% compared to the untreated controls and PDZ-PDT, respectively. Dual-photosensitizer PDT reduced the tumor thickness reduction by ≈75%, ≈70% and ≈50% compared to the control, PDZ-PDT and VIS-PDT groups, respectively. The addition of OCA gave greater enhancement with VIS-PDT than PDZ-PDT (*p* < 0.0001) and, when used with dual-agent PDT, resulted in complete tumor eradication within the 10 days observation period.

A summary of the key results in the intradermal model is presented in Table 1. In this study, the PDT outcome was assessed by OCT angiography, ultrasound/photoacoustic imaging and quantitative (immuno) histology. The metrics to identify the most effective PDT treatment protocol were reduction in the tumor thickness and volume, decrease in SGR and increase in DT values, and absence of S100 and Ki67 staining. Although some of these parameters were similar in different treatment cohorts, the only protocol that achieved all these metrics for pigmented (and non-pigmented) tumors was dual-agent PDT with optical clearing.

## 4. Discussion

Melanoma is an aggressive and highly lethal cancer that survives in a hypoxic environment and invades surrounding tissues [39,40]. If not diagnosed at an early stage, the morbidity and mortality rates are high despite the best current therapies. Especially when highly pigmented, melanoma has shown poor response to photodynamic therapy using established photosensitizers such as methylene blue (MB) [14], Visudyne [15], or aminolevulinic acid-induced protoporphyrin IX [41]. The poor responses achieved using commercially available photosensitizers motivated the development and testing of photosensitizers excited in the near infra-red and the use of tumor-targeting strategies. Although some studies demonstrated a degree of tumor control, none achieved complete tumor response in an immunodeficient model [42,43,44,45]. Here, we chose to investigate the effects of cellular PDT with glucamine-salt of chlorine e6 (Photodithazine, PDZ) [46,47] and vascular-PDT with Visudyne [37,48], since both are commercially available and have been used for a variety of non-oncological and oncological applications. 

PDZ-mediated cellular PDT showed poor responses in both cutaneous models, with only a slight reduction in tumor volume even in the non-pigmented tumors. OCT-angiography assessment of pigmented tumors in the window chamber model showed minimal vascular response immediately after PDZ-PDT and recovery of blood microvasculature within 2 h. Interestingly, the larger feeder vessels were completely destroyed at day 7 and replaced by a rich microvasculature network, preventing full tumor eradication. 

Vascular-PDT should, in principle, be effective for treating well-vascularized tumors such as melanoma, providing that the poor light penetration can be addressed. Here, VIS-PDT alone, which is predominantly vascular-mediated due to the short drug-time interval used, resulted in a strong response in the non-pigmented tumor. Not surprisingly, however, only partial response was observed in pigmented lesions, with regrowth in the tumor periphery. As also expected, the vascular response was more evident in the pigmented window chamber model for VIS-PDT than for PDZ-PDT, with progressive vascular damage and partial shutdown, together with blood leakage at 2–48 h after PDT. Nevertheless, the larger feeder vessels were re-established at day 7 and the tumor was not eradicated, despite there being growth delay. 

PDT treatment protocols, including in melanoma, are usually based on a single photosensitizer. Acedo et al. proposed combining two photosensitizers, zinc(II)-phthalocyanine (ZnPc) and a cationic porphyrin meso-tetrakis(4-N-methylpyridyl)porphine (TMPyP), to improve the PDT effect. A synergistic effect was seen in vitro in the three tumor cell lines studied (HeLa, HaCat, MCF-7) and tumor growth delay was also seen in vivo [49]. In the present study the combination of Photodithazine (PDZ)- and Visudyne (VIS)-mediated PDT likewise improved the response in the non-pigmented model, leading to minimal residual tumor within the 10-day observation period following treatment. However, in the pigmented cutaneous melanoma model, and despite the complete vascular destruction seen in the window chamber model, limited efficacy was observed, showing only reduced growth rate and histology revealed the continuing presence of S100 and Ki67, indicating active tumor-cell survival. If this incomplete response is due primarily to the limited light penetration in the pigmented intradermal model, then optical clearing should be beneficial. 

As predicted, but not previously reported, the effect of optical clearing on the efficacy of PDT was more marked in the pigmented than non-pigmented tumors. Indeed, minimal improvement in treatment response was seen in the latter, where both vascular- and dual-agent PDT were already effective in these ≈1 mm thick lesions. The relatively poor responses for non-pigmented tumors treated with cellular-PDT, with or without OCA, suggests that other factors such as photosensitizer efflux and/or defects in the apoptotic pathways may be involved, as identified by Baldea et al. [7] and Huang et al. [17]. We note that optical clearing may still be effective in thicker non-pigmented tumors. This is currently being investigated in a Phase I clinical trial of nodular basal cell carcinoma, in which no toxicity nor adverse effects on the established ALA-PpIX PDT responses have been seen in 14 lesions in 12 patients to date [50].

The positive effect of the optical clearing was substantial in the pigmented tumors, both for single- and, particularly, dual-agent PDT for which no tumor was detectable in vivo at 10 days post-treatment. This was supported by the negative S100 protein and Ki67 expression data. To our knowledge, this is the first demonstration of effective PDT treatment of pigmented melanoma in an immunocompromised mouse model. The fact that a dual-photosensitizer approach supplemented with OCA was needed to achieve complete response underscores the refractory nature of this tumor, but also suggests that its successful management is possible, albeit procedurally challenging. The high efficacy may translate into improved survival and this is currently under investigation. Additionally, we are evaluating two complementary strategies to enable effective treatment of even thicker lesions: (1) combining OCA with whitening agents that fragment the melanin granules, thereby further reducing the light attenuation [51] and (2) the use of micro-needle arrays to enhance OCA delivery to greater lesion depth [52].

In addition to the destruction of the primary tumor, PDT has immune-modulation/immunoregulatory effects, including stimulation of tumor-specific cytotoxic T-cells to destroy distant untreated tumor cells, thereby inhibiting tumor metastasis, and induction anti-tumor memory to alter tumor progression and prevent recurrence [53]. These immune effects are highly relevant in melanoma since surgery is the current first-line treatment and there is preclinical and clinical evidence that surgery-related stress can reduce immune system function in the perioperative period, leading to an increase in metastatic spread compared to non-surgical controls [54]. This has been demonstrated preclinically in melanoma, as well as in breast and lung tumors [54]. Pucelik and collaborators have investigated a nanoparticle-based photosensitizer for melanoma treatment using an immunocompetent mice model and demonstrated a complete response to up to 1 year after the PDT [16]. However, no histological measurements were performed and the contribution of possible immunological effects of the treatment were not evaluated. The possibility of using PDT to control primary melanoma, the feasibility of which we have demonstrated here, while also stimulating the immune system to reduce the tumor (metastatic) burden represents a potentially new paradigm for effective management of melanoma patients. The immunological effects of OCA-enabled, dual-photosensitizer PDT given as a stand-alone treatment and in combination with surgery are currently being explored. Moreover, if PDT does have significant immune effects in melanoma, it could also be used to complement other immunotherapy approaches [3].

## 5. Conclusions

PDT using conventional photosensitizers has not resulted in clinically useful responses in cutaneous melanomas, which are usually heavily pigmented. The preclinical in vivo experiments reported here support the hypotheses that using combined cellular- and vascular-PDT improves the therapeutic response compared with single-agent PDT and that optical clearing results in better tumor responses for both single- and dual-agent PDT. Complete tumor eradication in a cutaneous pigmented melanoma model by combining optical clearing with dual-agent PDT has been demonstrated for the first time. The next steps will be to optimize the combination PDT treatment and the OCA delivery and to assess the resulting impact on local control, survival and metastatic spread over longer follow-up periods. The translational potential of this novel strategy is that it could make PDT as effective in pigmented melanoma as in other skin tumors, which, given the refractory nature of this disease and its lack of effective treatments, would represent a significant advance.

## Figures and Tables

**Table 1:** Summary of the key results at 10 days after photodynamic therapy (PDT), using specific growth rate (SRG) and remaining-thickness metrics for tumor growth control. Ki-67 positive fraction <99% indicates fullthickness tumor eradication.

Group	OCA	Metrics	Non-Pigmented Melanoma	Pigmented Melanoma
PDZ-PDT	Without OCA	SGR reduced >80%	Yes	No
		Thickness <1 mm	No	No
		Ki-67 positive fraction <99%	No	No
	With OCA	SGR reduced >80%	Yes	No
		Thickness <1 mm	No	No
		Ki-67 positive fraction <99%	No	No
VIS-PDT	Without OCA	SGR reduced >80%	Yes	No
		Thickness <1 mm	Yes	No
		Ki-67 positive fraction <99%	Yes	No
	With OCA	SGR reduced >80%	Yes	Yes
		Thickness <1 mm	Yes	Yes
		Ki-67 positive fraction <99%	Yes	No
PDZ-PDT+ VIS-PDT	Without OCA	SGR reduced >80%	Yes	Yes
		Thickness <1 mm	Yes	No
		Ki-67 positive fraction <99%	Yes	No
	With OCA	SGR reduced >80%	Yes	Yes
		Thickness <1 mm	Yes	Yes
		Ki-67 positive fraction <99%	Yes	Yes

**Table 2:** Specific growth rates and doubling times at 10 d after PDT. * denotes statistical significance (*p* < 0.05) between treated groups and untreated controls and ** denotes statistical significance to treated groups and untreated controls and between treated groups with and without optical clearing agent (OCA).

Specific Growth Rate (SGR) and Doubling Time (DT)				
	Non-Pigmented		Pigmented	
	SGR (mm^3^/day)	DT (day)	SGR (mm^3^/day)	DT (day)
**Control**	0.50 ± 0.15	1.34 ± 0.35	0.68 ± 0.09	1.03 ± 0.13
**PDZ-PDT**	0.09 ± 0.04 *	9.90 ± 6.38	0.62 ± 0.08	1.12 ± 0.15
**OCA+PDZ-PDT**	0.13 ± 0.09 *	9.71 ± 10.10	−0.04 ± 0.09 **	−1.40 ± 12.32
**VIS-PDT**	0.11 ± 0.04 *	6.72 ± 2.88	0.32 ± 0.07 *	2.22 ± 0.49
**OCA+VIS-PDT**	0.02 ± 0.17 *	2.61 ± 5.60	−0.25 ± 0.10 **	−3.12 ± 1.36
**PDZ-PDT+VIS-PDT**	−0.15 ± 0.00 *	−4.79 ± 0.07	0.07 ± 0.02 *	10.15 ± 1.94
**OCA+(PDZ-PDT+VIS-PDT)**	−0.27 ± 0.001 *	−2.58 ± 0.001	−0.24 ± 0.01 **	−2.92 ± 0.46

## Supplementary Materials

The following are available online at https://www.mdpi.com/2072-6694/12/7/1956/s1, Figure S1: Histology with H&E, S100 and Ki-67 staining for the non-pigmented melanoma groups. Figure S2: Figure S1 at higher magnification. Figure S3: Histology with H&E, S100 and Ki-67 staining for the pigmented melanoma groups. Figure S4: Figure S3 at higher magnification
