# Supplementary material for: Dual-Agent Photodynamic Therapy with Optical Clearing Eradicates Pigmented Melanoma in Preclinical Tumor Models
Source: Cancers (Basel). 2020 Jul 18;12(7):1956. doi: 10.3390/cancers12071956 (PMC7409296; doi:10.3390/cancers12071956)
Supplement: Supplementary file 1 [file cancers-12-01956-s001.pdf]

SUPPLEMENTARY MATERIAL

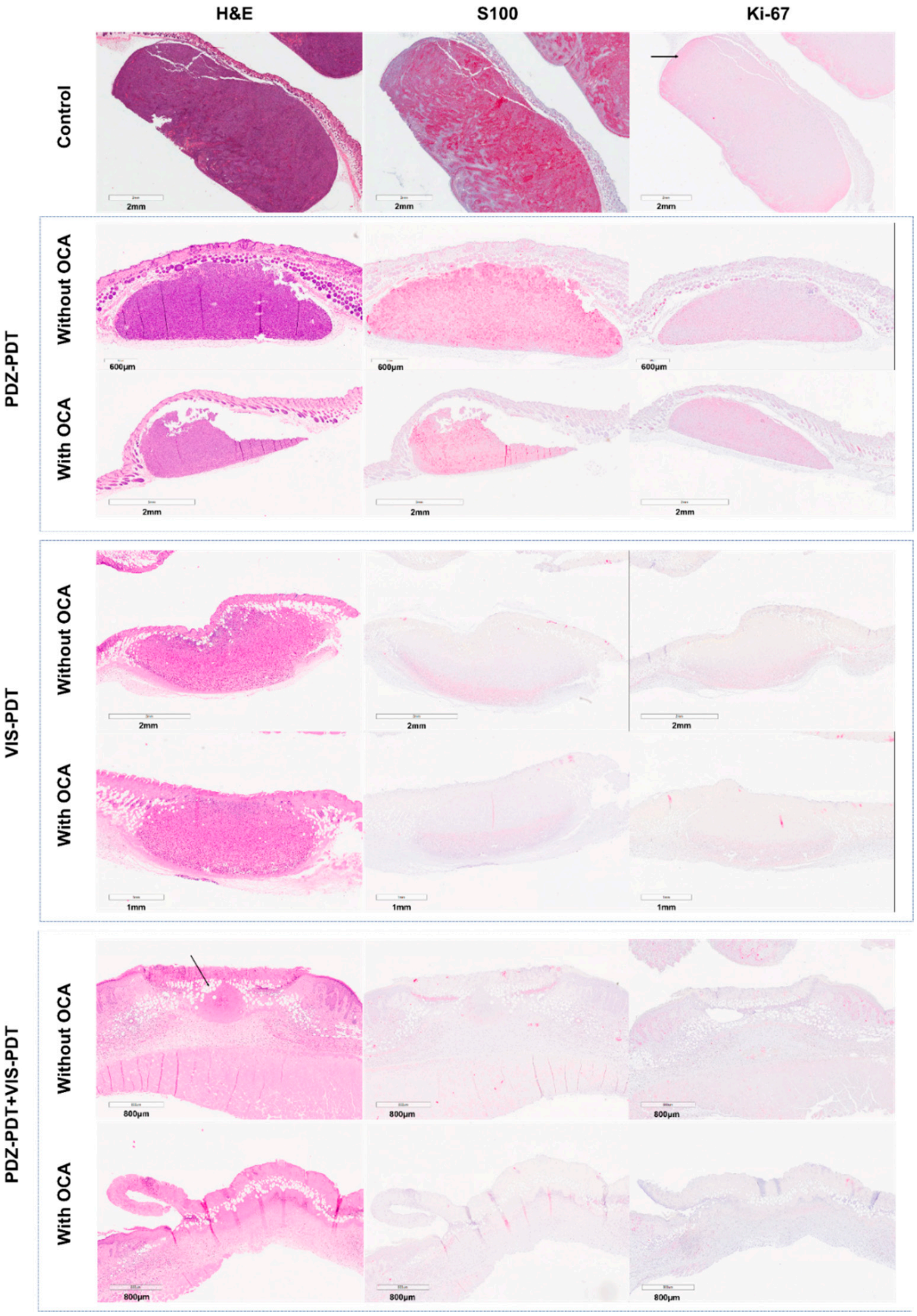

Figure 1. Representative examples of non-pigmented tumor before treatment, demonstrating proliferation at the tumor margin, as indicated by Ki-67 staining, with cell invasion in the superficial layers that (with or without pigmentation) is characteristic of this model. In the example of PDZ-PDT without OCA the superficial tumor layers are slightly damaged but S100 is still expressed throughout the entire thickness and 64% of the remaining cells are Ki-67 positive. With optical clearing, a thin layer of healed skin overlying S100-expressing tumor is seen, indicating that the treatment was only

superficial, and Ki-67 is positive for 53% of the cells. As expected with VIS-PDT, vascular responses are seen in the H&E sections, particularly at the tumor border, with necrosis in the deeper layers. Applying OCA resulted in some additional response, mainly in the superficial epidermal layer, likely due to improved homogeneity of the light distribution throughout the tumor volume. With VIS-PDT, minimal S100 and Ki-67 immunostaining is observed. The dual-photosensitizer treatment group without optical clearing shows a large necrotic area (arrow) and damage to the superficial layers of the tumor with H&E staining. No S100 or Ki-67 staining is apparent at any depth, as is also the case when optical clearing was used. The histology panel is shown in higher magnification in Supplemental Figure S2.

.

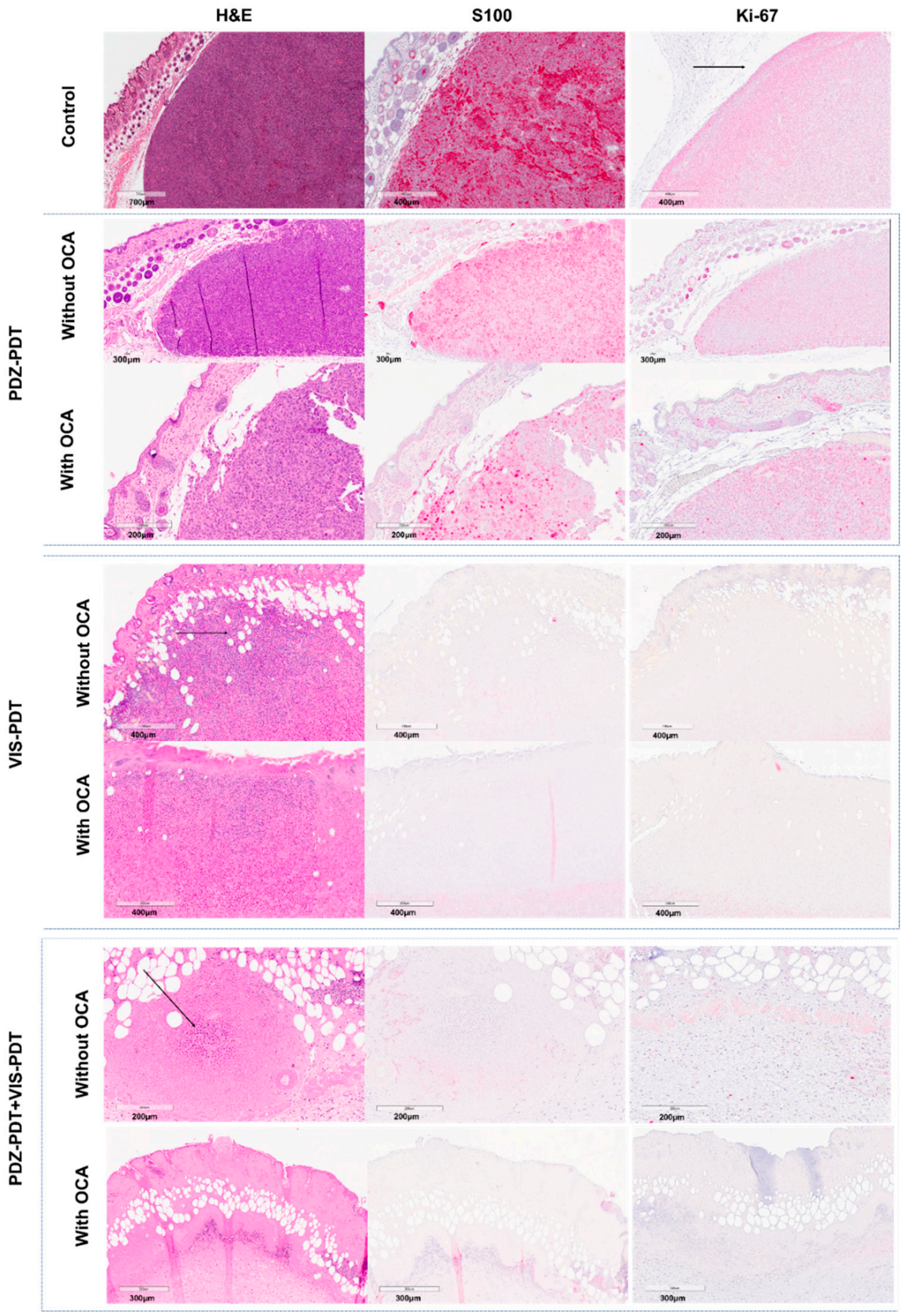

Figure 2. Histology of non-pigmented melanoma at 10 d after treatment. The untreated tumor shows S100 and Ki-67 staining throughout the entire thickness, with higher Ki-67 expression at the tumor borders (arrow). The PDZ-PDT group shows marked S100 and Ki-67 staining, covered by a normal skin layer of skin, suggesting that the treatment was able to treat only the surface layer of the tumor. The addition of OCA did not change the protein expression, but the tissue became more fragile. Vascular damage is seen in the VIS-PDT groups and there is no S100 and Ki-67 staining, with or without optical clearing. The same result is seen with dual-agent PDT. .

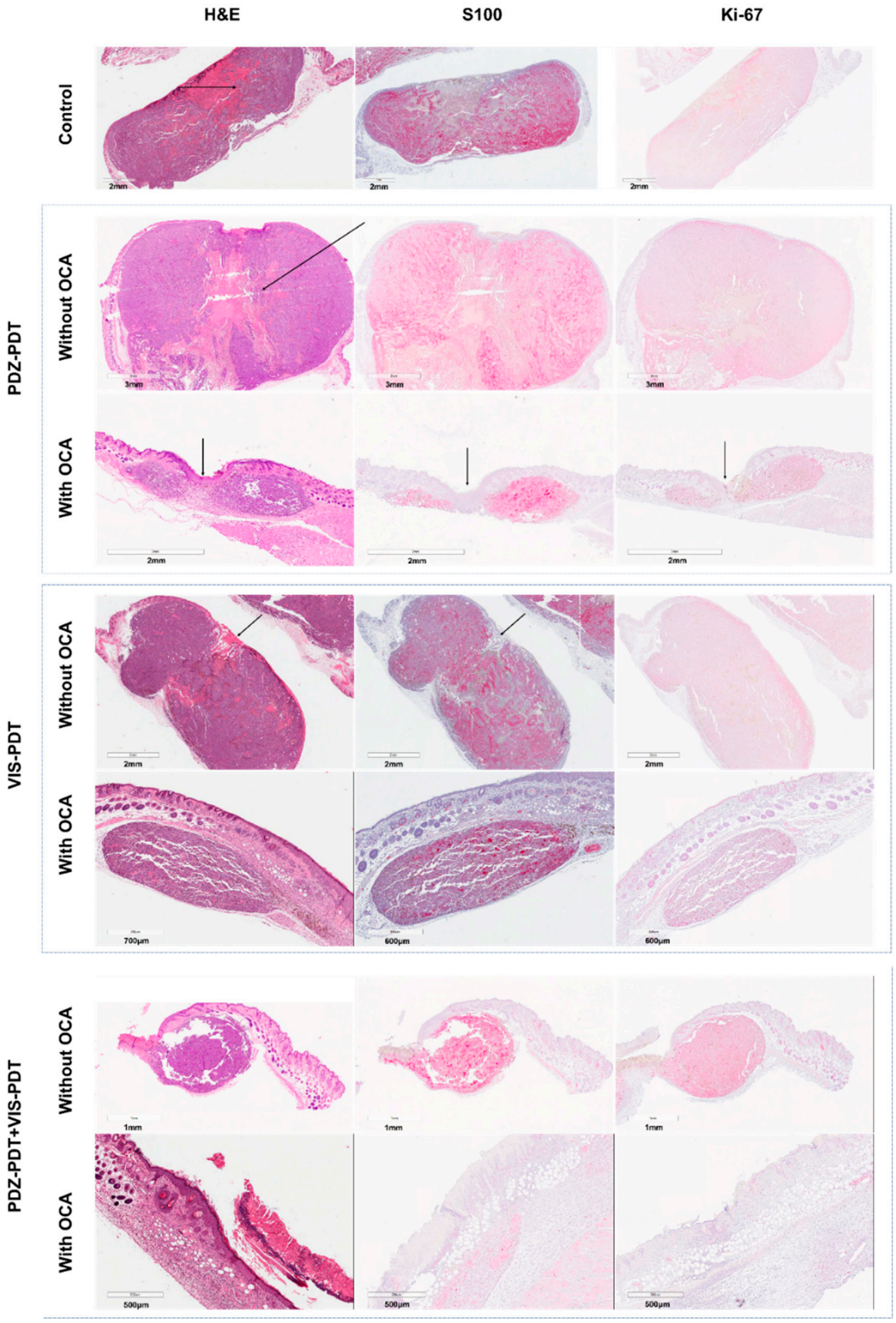

Figure 3. Examples of corresponding responses in pigmented tumors. Melanin is seen throughout the untreated tumor, as are the necrotic core (arrow), red blood cell release and adjacent tissue shrinking. The PDZ-PDT group shows a large superficial necrotic area, with epidermal loss, to up to 2/3 of the tumor thickness (arrow), beyond which viable tumor with high S100 protein and Ki-67 expression is present. Optical clearing significantly improved the response, as evidenced by the reduction in viable tumor thickness. Melanophages and a regeneration area are present in the tumor core (arrow), with absence of S100 and Ki-67 staining, although the tumor margins still expresses S100 and Ki67. VIS-PDT resulted in a large necrotic area on the surface of tumor, with ulceration in the inner epidermis (arrow), although there are still S100 and Ki-67 expressing tumor cells beneath and lateral of this region, confirming that the partial treatment response was due to the limited light penetration. Vascular damage and loss of epithelium, with collagen reorganization, is seen in the superficial tumor layers. With the addition of OCA, the remaining viable tumor, marked by positive S-100 and Ki67

staining, was thinner, consistent with improved light penetration. Vascular damage is also seen, mainly in the tumor periphery.

In the absence of optical clearing, dual-agent PDT improved the tumor responses compared to single-agent PDT, but this was not enough to treat the full tumor thickness, with S100 staining seen throughout the tumor and Ki-67 positivity in 18% of the remaining cells. Finally, however, the addition of OCA in the dual-agent treatment markedly enhanced the PDT response of the pigmented tumors, with no residual tumor detected with any of the histological stains. Higher magnification images are shown in Supplementary Figure 4.

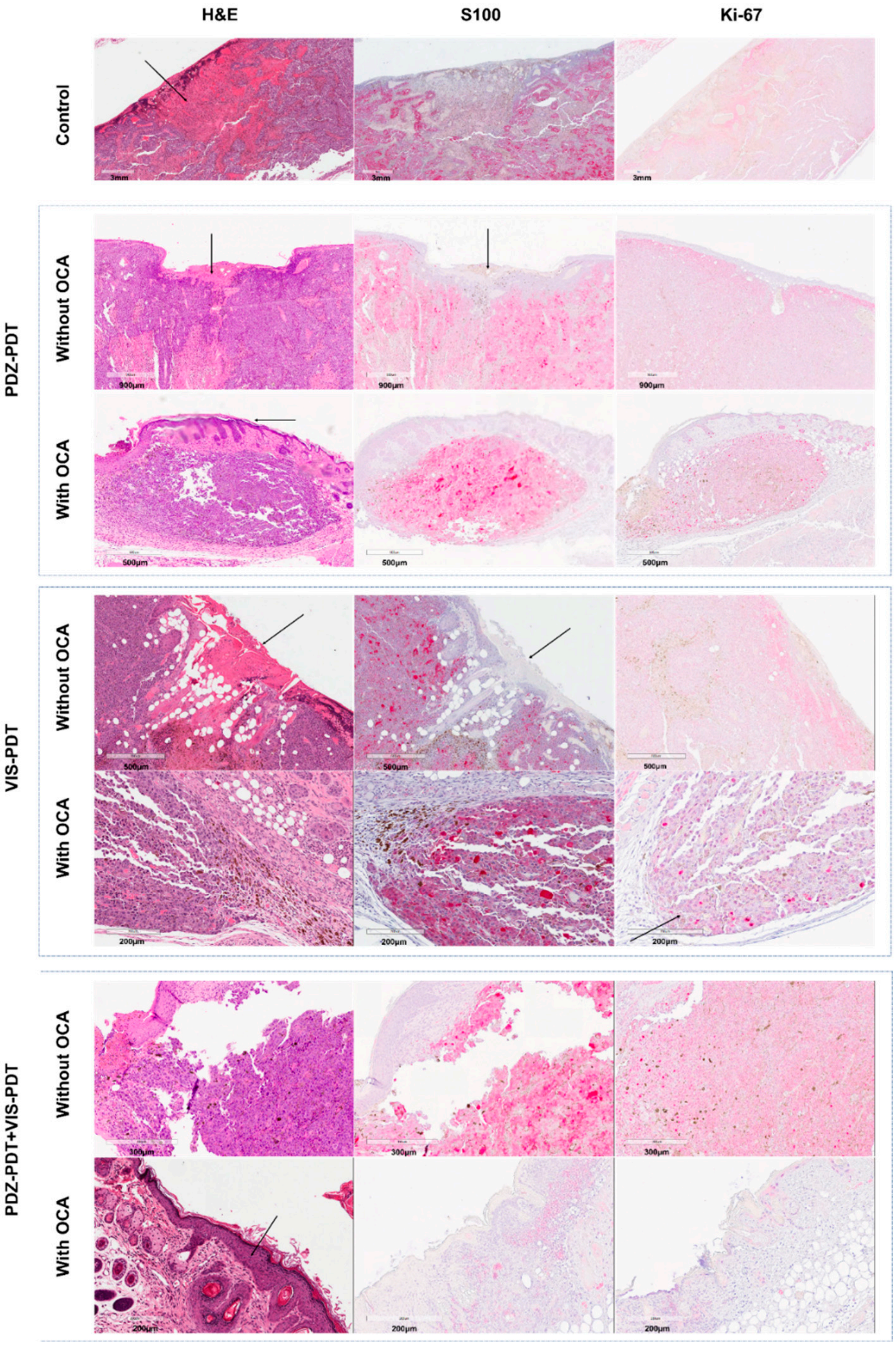

Figure 4. Histology of pigmented melanoma at 10d after PDT treatment. The untreated tumor shows a large necrotic core (arrow), with S100 and Ki-67 expression mainly in the tumor borders, Damage is seen in the superficial tumor layers in the PDZ-PDT group and the addition of OCA resulted in a more homogeneous outcome, but strong Ki-67 staining is seen in the tumor border. The tumor became more fragile with the dual-agent PDT, but S100 and Ki-67 is still expressed throughout the entire tumor. Dual-agent combined with PDT eradicated the tumor, so that no S100 or Ki-67 staining is observed. .
